# Role of Astroglial Hemichannels and Pannexons in Memory and Neurodegenerative Diseases

**DOI:** 10.3389/fnint.2016.00026

**Published:** 2016-07-20

**Authors:** Juan A. Orellana, Mauricio A. Retamal, Rodrigo Moraga-Amaro, Jimmy Stehberg

**Affiliations:** ^1^Departamento de Neurología, Escuela de Medicina, Pontificia Universidad Católica de ChileSantiago, Chile; ^2^Centro de Fisiología Celular e Integrativa, Facultad de Medicina, Clínica Alemana Universidad del DesarrolloSantiago, Chile; ^3^Laboratorio de Neurobiología, Centro de Investigaciones Biomédicas, Universidad Andres BelloSantiago, Chile

**Keywords:** connexin hemichannels, Cx hemichannels, connexin 43, pannexin, astrocytes, memory

## Abstract

Under physiological conditions, astroglial hemichannels and pannexons allow the release of gliotransmitters from astrocytes. These gliotransmitters are critical in modulating synaptic transmission, plasticity and memory. However, recent evidence suggests that under pathological conditions, they may be central in the development of various neurodegenerative diseases. Here we review current literature on the role of astroglial hemichannels and pannexons in memory, stress and the development of neurodegenerative diseases, and propose that they are not only crucial for normal brain function, including memory, but also a potential target for the treatment of neurodegenerative diseases.

## Connexin Hemichannels and Pannexin Channels

In the 90’s, a handful of studies demonstrated that molecular and ionic interchange between the intracellular and extracellular compartments can occur via a family of plasma membrane channels called hemichannels (Goodenough and Paul, 2003). Originally known as the building blocks of gap junction channels (GJC; Revel and Karnovsky, 1967), hemichannels release relevant quantities of autocrine and paracrine signaling molecules (e.g., ATP, glutamate, NAD^+^ and PGE_2_) to the extracellular milieu, as well as the influx of small molecules and ions of up to ~1.5 kDa (e.g., glucose, cADPR and Ca^2+^) (Bruzzone et al., 2001; Stout et al., 2002; Ye et al., 2003; Cherian et al., 2005; Retamal et al., 2007; Song et al., 2011; Fiori et al., 2012). Each hemichannel or connexon is comprised of six connexins (Cxs). Cxs encompass a highly conserved protein family encoded by 21 genes in humans and 20 in mice, with orthologs in other vertebrate species (Eiberger et al., 2001; Abascal and Zardoya, 2013). Recently, another gene family encoding a set of three membrane proteins, termed pannexins (Panx 1-3), was identified (Panchin et al., 2000). Pannexins form single plasma membrane channels (Bruzzone et al., 2003) termed pannexons that participate in paracrine and autocrine signaling among cells (Bao et al., 2004; Locovei et al., 2006a).

Several studies show that hemichannels and pannexons play different physiological roles in the central nervous system (CNS), including ischemic tolerance (Orellana et al., 2014), establishment of adhesive interactions (Cotrina et al., 2008), fear memory consolidation (Stehberg et al., 2012), synaptic transmission (Prochnow et al., 2012; Chever et al., 2014), spontaneous electrical activity (Moore et al., 2014), glucose sensing (Orellana et al., 2012), chemoception (Reyes et al., 2014), blood-brain barrier (BBB) permeability (De Bock et al., 2011), redox sensing (Retamal et al., 2006) and neuronal migration (Liu et al., 2012).

## Hemichannels and Pannexons in Astrocytes

Astrocytes play key roles in CNS function by providing nutrients (e.g., lactate; Pellerin, 2008) and redox molecules (Wilhelm and Hirrlinger, 2012), maintaining ionic balance (Kimelberg, 2005), K^+^ clearance mediated by Na^+^/K^+^-ATPase (Sibille et al., 2014), glucose and lactate metabolism (Allaman et al., 2011), and neurotransmitter recycling of the two most abundant neurotransmitters in the brain: glutamate and GABA (Simard and Nedergaard, 2004). They also regulate cerebral microcirculation (Takano et al., 2006), and BBB permeability (Alvarez et al., 2013), among many other roles essential for normal brain function.

**Figure 1:**
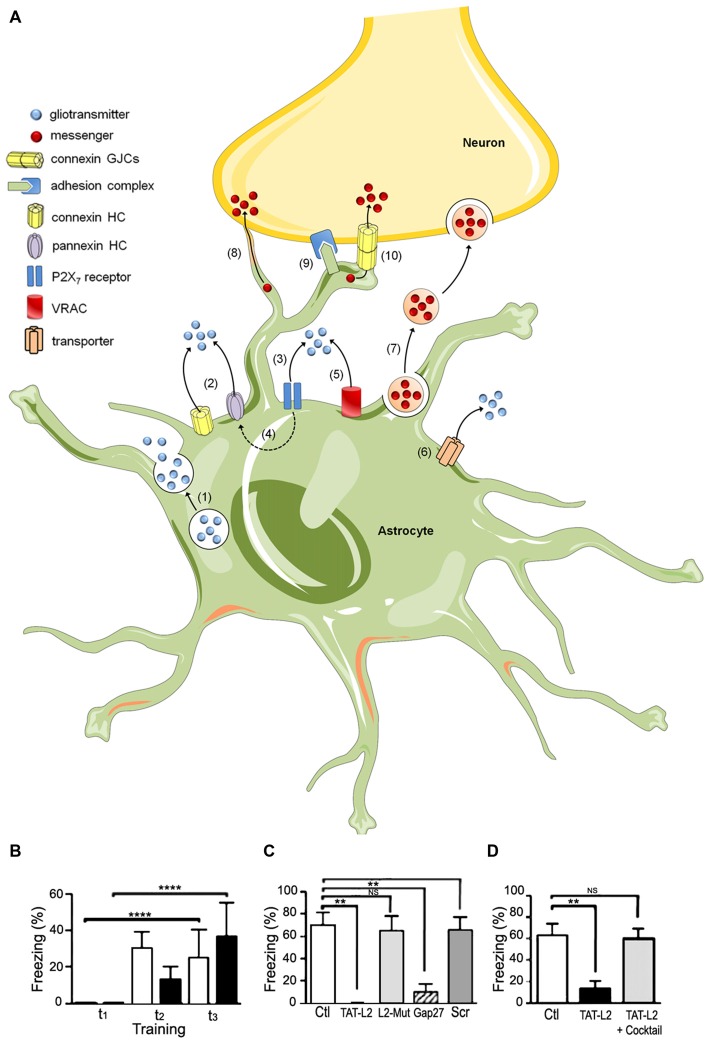
**The tripartite synapse; hemichannels, pannexons and their role in memory consolidation. (A)** Astrocytes release gliotransmitters (e.g., glutamate, D-serine and ATP) through Ca^2+^-dependent exocytosis (1) and opening of connexin (Cx) and pannexin (Panx) hemichannels (2). Long-lasting activation of P2X7 by ATP may lead to large currents and release of gliotransmitters (3), effect that may be mediated by Panx1 hemichannels (4). Gliotransmitter release may also occur through volume-regulated anion channels (VRAC) (5) and different carriers and/or co-transporters acting normally or in reverse (6) (e.g., excitatory amino-acid transporters, the cysteine-glutamate antiporter and the D-serine/chloride co-transporter). Astrocytes can also communicate with neurons via the release of vesicles (e.g., exosomes, microparticles and apoptotic bodies), containing different cellular messengers (e.g., mRNA, viruses and organelles) (7). Adjacent glial cells and neurons can communicate directly through F-actin-based transient tubular connections known as tunneling nanotubes (8), via cell-to-cell contacts between membrane-bound ligand molecules and their receptors (9) or intercellular channels known as gap junctions (10). **(B–D)** Blockade of astroglial Cx43 hemichannel opening in the basolateral amygdala by intra-BLA microinjection of TAT-L2 mimetic peptide had **(B)** no effect in short term fear conditioning memory, **(C)** but blocked fear memory consolidation as assessed 24 h after training. This amnesic effect was also found after injection of the more unspecific hemichannel blocker GAP27, but was absent when a scrambled peptide was used (scr) or a similar peptide to TAT-L2 with two aminoacids mutated (L2-mut) to interfere with its affinity for Cx43. **(D)** A minimal dose of TAT-L2 still capable of blocking memory consolidation (TAT-L2) was co-injected into the basolateral amygdala with a mixture of TAT-L2 and various putative gliotransmitters (TATL2 + cocktail), including glutamate, D-serine, glycine, ATP, etc. The microinjection of the mixture prevented the amnesic effects of intra-amygdalar TAT-L2 peptide (reprinted from Stehberg et al., 2012 with permission). *P < 0.05; **P < 0.01; ***P < 0.001; ****P < 0.0001.

Additionally, it has been proposed that astrocytes actively participate in neuronal transmission and synaptic plasticity (Barres, 1989; Nedergaard, 1994; Parpura et al., 1994), via the release of molecules, known as gliotransmitters, into the synaptic cleft. In this context, Araque et al. (1998a,b) found that astrocytes surround synaptic buttons and release molecules into the synaptic cleft, modulating both pre- and post-synaptic activity. The term “tripartite synapse” was coined to describe synapses between neurons and astrocytes (Araque et al., 1999). Henceforth, several studies have proposed different pathways of gliotransmitter release from astrocytes, which appear to act in parallel and include P2X7 receptors (Duan et al., 2003), pannexons (Iglesias et al., 2009), Cx43 hemichannels (Cotrina et al., 1998), transporters (Szatkowski et al., 1990), and vesicles (Parpura et al., 1994). For a summary of main gliotransmitter release mechanisms see Figure 1A.

Astrocytes show the highest level of Cx expression among brain cells, with Cx43 being the most abundantly expressed both *in vitro* and *in vivo* (Dermietzel et al., 1991; Naus et al., 1991). Astrocytes also express Cx30 (Nagy et al., 1999), Cx26 (Rash et al., 2001), and may also show Panx1 (Iglesias et al., 2009), at least after stress (Orellana et al., 2015). Cx43 and Panx1 form functional hemichannels and pannexons in astrocytes *in vitro* and *ex vivo* (Contreras et al., 2002; Iglesias et al., 2009; Orellana et al., 2011a).

Embedded in the “tripartite synapse”, astrocytes express a plethora of receptors (reviewed in Moraga-Amaro et al., 2014) and respond locally to neurotransmitters through the above mentioned mechanisms of gliotransmitter release, including the activation of hemichannels and pannexons (Malarkey and Parpura, 2008). Indeed, several gliotransmitters such as D-serine, glutamate, ATP and lactate have been reported to be released via astrocytic hemichannels (Stout et al., 2002; Ye et al., 2003; Karagiannis et al., 2015) or pannexons (Iglesias et al., 2009; Pan et al., 2015) *in vitro*. This gliotransmitter release has been proposed to be necessary for different CNS functions *in vivo* (Orellana and Stehberg, 2014; Montero and Orellana, 2015). Other *in vitro* studies have reported Cx43 hemichannels to be permeable to NAD^+^ (Bruzzone et al., 2001), glucose (Retamal et al., 2007), taurine (Stridh et al., 2008), and Ca^2+^ (Schalper et al., 2010). Moreover, given that GJCs have been shown to allow for the passage of small peptides with a molecular weight of up to 1.8 kDa (Neijssen et al., 2005) and short interfering RNAs (Valiunas et al., 2005), it is possible that hemichannels may also allow the passage of such molecules, hypothesis that has not been tested so far.

Most early studies on hemichannels were performed *in vitro*, and suggested that Cx43 hemichannels have a low open probability in physiological conditions, requiring depolarized membrane potentials as high as +60 mV. Given that astrocytes are considered non-excitable cells in terms of membrane potential, their opening under physiological conditions was considered virtually impossible. However, later studies showed hemichannel opening at negative membrane potentials as measured by hemichannel-mediated dye uptake and ionic currents (Contreras et al., 2003; Retamal et al., 2007).

Recent *in vitro* studies have shown that astroglial Cx43 hemichannel activity changes in response to general anesthetics (Liu et al., 2016) antidepressants (Jeanson et al., 2015) and modafinil (Duchêne et al., 2016), suggesting that they may also be drug targets.

## Evidence for Astroglial Hemichannel Function *In Vivo*

The evidence of a role for astroglial hemichannels and pannexons *in vivo* in astroglial physiology and CNS function is much more limited, and is only now beginning to emerge. A recent study reported that astroglial Cx43 hemichannels are active in hippocampal slices during basal conditions and that astroglial Cx43 hemichannel-dependent release of ATP increases basal excitatory (glutamatergic) synaptic transmission through P2 receptors (Chever et al., 2014). Similar results were reported in neurons that project to the vagal nerve (Retamal et al., 2014).

Astroglial Cx43 hemichannel opening may also contribute to neuronal oscillations. Roux et al. (2015) reported that astroglial Cx43 hemichannel opening in olfactory bulb slices increases the amplitude of slow oscillations in mitral cells, affecting their firing rate. Hemichannel activity is also necessary for maintaining spontaneous activity in the cortex during development (Moore et al., 2014). It remains unknown whether hemichannels are still relevant for spontaneous activity in the adult cortex.

Yet another example of the role of astroglial hemichannels in CNS function measured *in vivo* can be found in the retrotrapezoid nucleus, in which the firing rate of CO_2_/H^+^-sensitive neurons acting as chemoreceptors (Wenker et al., 2012; Reyes et al., 2014) is modulated by ATP release from astrocytes via Cx26 hemichannels (Huckstepp et al., 2010).

In a recent study by Orellana et al. (2015), we reported from *ex-vivo* hippocampal slices that acute 2 h restraint stress in mice induces opening of astroglial Cx43 hemichannels, while chronic 10-day immobilization stress—a model used to induce depression in rodents—leads to increased opening of Cx43 hemichannels, and recruitment of astroglial Panx1 channels. This increase in hemichannel activity correlated with an increase in glutamate and ATP release, being dependent on glutamatergic N-methyl-D-aspartate (NMDA) and purinergic receptor signaling (Orellana et al., 2015). Moreover, Garré et al. (2016) showed that FGF-1 promotes inflammatory responses in acute spinal cord slices by a mechanism that involves release of ATP through Panx1 channels. Finally, in another study we shall discuss in more detail later, we showed that Cx43 hemichannels are necessary for fear memory consolidation in the basolateral amygdala (Stehberg et al., 2012).

As can be deduced from the above paragraph, *in vivo* evidence supporting a role for hemichannels and pannexons in CNS function is very recent, still limited in number but growing fast.

Astroglial hemichannels open in response to local increments in intracellular Ca^2+^. Astrocytes express receptors and respond to most neurotransmitters known to be relevant for memory (for a review see Moraga-Amaro et al., 2014) via fast and local Ca^2+^ oscillations or inter-astrocytic Ca^2+^ waves at speeds matching neuronal activity (Winship et al., 2007). Thus, astroglial activation may trigger the opening of hemichannels or pannexons and the concomitant release of D-serine, glutamate, ATP and lactate, among various other gliotransmitters (Orellana and Stehberg, 2014). D-serine is a co-agonist of NMDA receptors critical for synaptic plasticity (Henneberger et al., 2010). Glutamate is the main excitatory neurotransmitter in the CNS and lactate is critical for brain metabolism and acts as a neurotransmitter activating NMDA receptors (Yang et al., 2014), all of which are critical for synaptic plasticity and memory. ATP mediates propagation of inter-astrocytic Ca^2+^ waves by activating astroglial purinergic receptors, whereas its conversion into adenosine may activate neuronal purinergic receptors (Zhang et al., 2003). As a consequence, it is likely that these functions are mediated by astroglial hemichannels and pannexons, but direct *in vivo* evidence is still lacking.

## Astroglial Hemichannels and Pannexons in Memory

As mentioned earlier, astroglial hemichannels and pannexons allow for the delivery of a wide variety of gliotransmitters to the extracellular milieu. However, the role of these channels in brain function under physiological conditions, and particularly in memory, has only recently begun to be elucidated. In 2012, we demonstrated that blockade of Cx43 hemichannels at the basolateral amygdala *in vivo*, using a mimetic peptide known as TAT-L2, had no effects on short term memory (Figure 1B), yet blocked memory consolidation, inducing amnesia for auditory fear conditioning 24 h after training (Figure 1C). Interestingly, the amnesic effect of the peptide was prevented by co-injecting it together with a cocktail of gliotransmitters, including glutamate, D-serine, glycine, lactate, ATP and glutamine (Figure 1D; Stehberg et al., 2012). This indicates that the opening of Cx43 hemichannels permits the release of gliotransmitters necessary for memory consolidation, but we were not able to identify the gliotransmitter or combination of gliotransmitters that is critical for memory. In this respect, a previous study by Henneberger et al. (2010) reported that preventing calcium oscillations in astrocytes averted long-term potentiation (LTP, a model of synaptic plasticity associated to memory) in hippocampal slices. This effect was reverted by exogenous administration of D-serine to the preparation (Henneberger et al., 2010). D-serine is a co-agonist of glutamate NMDA receptors which is secreted by astrocytes and is critical for LTP induction (Henneberger et al., 2010; Kang et al., 2013). There is currently no direct evidence that D-serine can be released via Cx43 hemichannels, but it is possible, as astroglial pannexons have been reported to release D-serine *in vitro* (Pan et al., 2015).

Genetic studies affecting Cx expression have had limited value in deciphering the role of hemichannels, as current genetic approaches affect the expression of both hemichannels and GJCs and do not allow for the distinction of the effects of either. Double knockout mice for both Cx43 and Cx30, the two main Cxs expressed in astrocytes, show enhanced synaptic transmission, attenuated LTP and increased long-term depression (LTD) in hippocampal CA1 pyramidal cells (Pannasch et al., 2011), which are critical for memory formation.

It is still debated whether Pannexons form GJCs *in vitro* (Sosinsky et al., 2011; Sahu et al., 2014). Unlike Cx43, which is expressed mainly in astrocytes (also reported in microglia, radial glia and neural progenitors; Nadarajah et al., 1997; Boucher and Bennett, 2003), Panx1 is expressed in both astrocytes and neurons (Zoidl et al., 2007; Iglesias et al., 2009; Santiago et al., 2011). Thus, Panx1 deficiency by genetic knockout or pharmacological approaches cannot distinguish neuronal from astroglial pannexons. Both pharmacological blockade and genetic deficiency of Panx1 channels induce increased excitability, enhanced LTP, reduced LTD and impairments in object recognition and spatial memory (Prochnow et al., 2012; Ardiles et al., 2014). This evidence depicts a clear role for Panx1 in synaptic plasticity and memory, regardless of whether they originate from astrocytes, neurons, or both.

## Hemichannels and Pannexons in Psychiatric Disorders Associated with Cognitive Dysfunction

Thus far no study has reported a direct role for astroglial hemichannels in psychiatric disorders that can be associated with memory. However, in Orellana et al. (2015), we showed that astroglial Cx43 hemichannel activity in the hippocampus is increased after acute restraint stress in mice, effects that were associated with a Cx43-dependent increase in extracellular levels of glutamate and ATP (Orellana et al., 2015). Interestingly, when mice underwent a protocol of chronic restraint stress commonly used to induce depressive-like symptoms in mice, an even greater increase in Cx43 hemichannel activity was induced, together with Panx1 channel opening, with the concomitant Cx43- and Panx1-dependent release of glutamate and ATP (Orellana et al., 2015). This study was followed by the work of Quesseveur et al. (2015), reporting that knockdown of Cx43 in mice induced anxiolytic- and antidepressant-like effects and an increase in freezing in the fear-conditioning paradigm. Interestingly, it was found that chronic corticosterone administration (another model used to induce depression in rodents), caused an increase in the expression of phosphorylated Cx43 in the hippocampus, effect that was reversed by successful antidepressant treatment (Quesseveur et al., 2015). This is further supported by a recent study showing that antidepressants affect astroglial Cx43 GJC and hemichannel activity (Jeanson et al., 2015). The above findings suggest that hippocampal Cx43 hemichannel activity may be important in stress responses and for the pathophysiology of depression. How they may contribute to arousal-induced memory enhancements, post-traumatic stress disorder or depression-associated cognitive impairments are exciting questions that may be answered in the near future.

## Hemichannels and Pannexons in Neurodegenerative Diseases

Many neurodegenerative diseases are characterized by destruction of memory related areas, including the hippocampus, prefrontal cortex, mediotemporal lobe, nucleus basalis, basal ganglia, etc. (for reviews on areas involved in memory see Packard and Knowlton, 2002; Jeong et al., 2015). For example, in Alzheimer’s disease (AD), extensive damage to the hippocampus and cortical areas has been associated with cognitive deficits (reviewed in Pini et al., 2016). Likewise, in frontotemporal dementia, damage to frontal and anterior temporal lobes was also associated with cognitive deficits (Finger, 2016), while in Parkinson’s disease (PD), damage to basal ganglia and frontal connectivity has also been correlated with cognitive deficits (Zgaljardic et al., 2003).

Various studies have linked dysregulation of hemichannel and pannexon permeability and expression, with the progression of different neurodegenerative diseases (Orellana and Stehberg, 2014; Penuela et al., 2014). However, the mechanisms by which astroglial hemichannels and pannexons contribute to neuronal damage remain elusive. It is possible that enhanced reactive astrogliosis evoked by neuroinflammation may alter different astroglial functions necessary for proper astrocyte-to-neuron crosstalk and neuronal survival, including synaptic gliotransmission, Ca^2+^ and NO signaling, as well as antioxidant and inflammatory responses. Hemichannels and pannexons are both affected by multiple inflammatory mediators released by reactive astrocytes and microglia (e.g., cytokines, NO and ROS; Retamal et al., 2007; Abudara et al., 2015). Inflammatory conditions could increase astroglial hemichannel/pannexon opening, leading to an uncontrolled influx of potentially toxic agents, as is the case of Ca^2+^. Because hemichannels are permeable to Ca^2+^ (De Bock et al., 2012; Fiori et al., 2012), and their opening is controlled by intracellular Ca^2+^ (De Bock et al., 2012), it is possible that overactivation of hemichannels/pannexons results in intracellular Ca^2+^ overload and the subsequent impairment of vital functions for astroglial survival; including energy metabolism, Ca^2+^ handling, osmotic regulation and antioxidant defense. Consistent with this notion, hemichannel and pannexon activity has been linked to an alteration in Ca^2+^ dynamics and cell death in reactive astrocytes (Orellana et al., 2010; Abudara et al., 2015; Rovegno et al., 2015). In addition, osmotic and ionic imbalances induced by uncontrolled influx of Na^+^ and Cl^−^ through these channels could result in further cell swelling and plasma membrane breakdown. Given that astrocytes provide metabolic, synaptic and trophic support to neurons and maintain the extracellular microenvironment, astroglial cell damage or dysfunction associated with hemichannel and pannexon opening could increase neuronal susceptibility to different pathological conditions (Contreras et al., 2004; Orellana et al., 2009).

**Figure 2:**
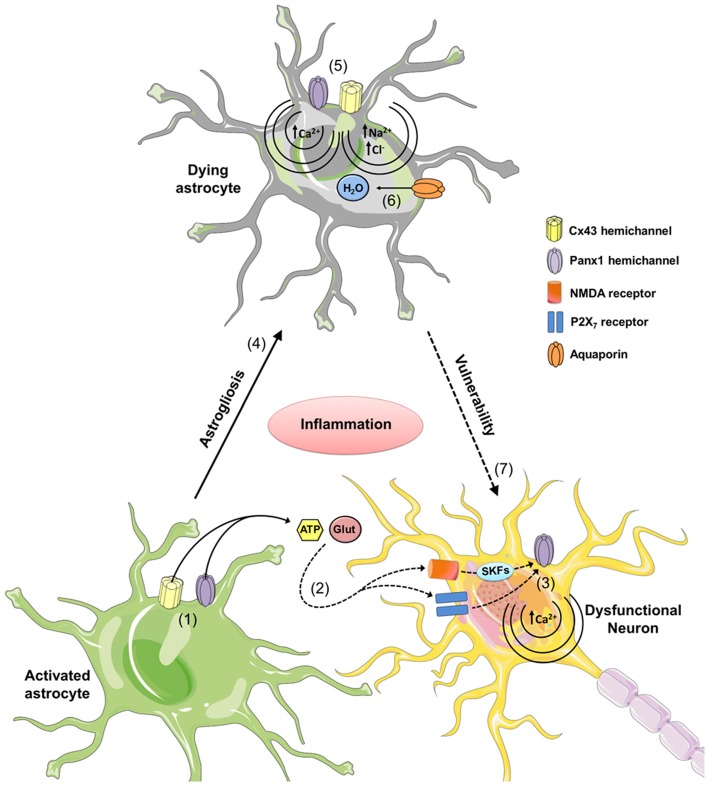
**The role of astrocytic hemichannels and pannexons during neurodegeneration.** During the early stages of various neurodegenerative diseases, increased inflammation opens astrocytic Cx43 hemichannels and Panx1 channels (1). This results in the release of the gliotransmitters ATP and glutamate, and increases activation of neuronal NMDA and P2X7 receptors (2). It is hypothesized that NMDA and P2X7 receptor activation increases the opening of neuronal Panx1 channels through phosphorylation of Panx1 by Src family kinases (SFKs) and direct protein-to-protein interactions, respectively (3). These P2X7-related protein interactions could affect intracellular Ca^2+^ homeostasis resulting in cell death. Uncontrolled activation of astrocytes may result in reactive astrogliosis and further cell death by a mechanism related to the opening of connexons and pannexons (4). In particular, dysregulated opening of Cx43 and Panx1 channels could elicit cellular damage by different mechanisms. At one end of the connexon, the entry of Ca^2+^ via the Cx43 hemichannels or Panx1 channels. The added Ca^2+^ activates phospholipase A_2,_ thus generating arachidonic acid and activating the cyclooxygenase and lipoxygenase pathways, resulting in increased free radicals, lipid peroxidation and plasma membrane damage (5). At the other end of the connexon, Na^+^ and Cl^−^ entry via Cx43 hemichannels or Panx1 channels may trigger cellular swelling due to an increased influx of H_2_O via aquaporins (6). Finally, given that astrocytes provide support to neurons, astroglial cell damage associated with hemichannel/pannexon opening could indirectly increase neuronal susceptibility and vulnerability due to the homeostatic imbalance occurring during neurodegeneration.

Alternatively, uncontrolled opening of hemichannels and pannexons induced by inflammatory conditions may trigger excessive release of molecules at toxic levels, such as glutamate and ATP. Consistent with this idea, astrocytes stimulated with amlyloid-β (Aβ) peptide exhibit increased Cx43 hemichannel-dependent release of glutamate and ATP, which are toxic for hippocampal and cortical neurons (Orellana et al., 2011a). Similarly, a follow-up work showed that astrocytes pre-treated with conditioned media from Aβ peptide-stimulated microglia release neurotoxic amounts of glutamate and ATP via Cx43 hemichannels when subjected to hypoxia in high glucose conditions (Orellana et al., 2011b). Interestingly, their release reduced neuronal survival via activation of neuronal NMDA/P2X7 receptors and Panx1 channels in neurons (Orellana et al., 2011a,b). Neurons express functional hemichannels formed by Cx36 and pannexons formed by Panx1 (Thompson et al., 2006; Zappalà et al., 2006), and the opening of Panx1 channels could occur via protein-protein interactions with activated P2X7 receptors (Iglesias et al., 2008), through increases in intracellular Ca^2+^ or phosphorylation triggered by activation of P2Y (Locovei et al., 2006b) or NMDA receptors (Weilinger et al., 2012). For a scheme of proposed roles for hemichannels and pannexons in neurodegeneration, see Figure 2.

## Concluding Remarks

Exciting research on astrocytes and particularly on astroglial hemichannels and pannexons characterizes the last few years. Although hemichannels and pannexons initially appeared to be one of the many cellular mechanisms used by astrocytes to share their gliotransmitters, accumulating evidence indicates that astroglial hemichannels play a key role in brain function under physiological conditions, and in pathology. In normal conditions astroglial hemichannels and pannexons are important for memory consolidation, stress response, and possibly even for the pathophysiology of depression. Given their role in NMDA-dependent plasticity, they may also prove to be relevant in depression-associated memory impairments. Yet in pathological conditions, they appear to have a central role in the development of neurodegenerative diseases. Although many questions remain unanswered regarding their role in memory and in cognitive dysfunction, it is clear that astroglial hemichannels and pannexons play essential roles, in sickness and in health, until death do us part.

## Author Contributions

Review was divided into equal parts, which were combined and edited by JS. JAO made Figure 2; JS and JAO made Figure 1. All authors listed, have made substantial, direct and intellectual contribution to the work, and approved it for publication.

## Funding

This work was supported by grants FONDECYT 11121133 (JAO), 1160710 (JAO), 1120214 (MAR) and 1160986 (JS); UNAB DI-603-14/N (JS), CORFO 14IDL2-30195 (JS); CAEN from the ISFN (JAO), and CONICYT ACT 1104 (MAR) and ACT 1411 (JAO). We wish to thank Kathleen McBennett for her contributions.

## Conflict of Interest Statement

The authors declare that the research was conducted in the absence of any commercial or financial relationships that could be construed as a potential conflict of interest.

## References

[B1] Abascal F. Zardoya R. (2013). Evolutionary analyses of gap junction protein families. Biochim. Biophys. Acta 1828, 4–14. 10.1016/j.bbamem.2012.02.007 22366062

[B2] Abudara V. Roux L. Dallérac G. Matias I. Dulong J. Mothet J. P.. (2015). Activated microglia impairs neuroglial interaction by opening Cx43 hemichannels in hippocampal astrocytes. Glia 63, 795–811. 10.1002/glia.22785 25643695

[B3] Allaman I. Bélanger M. Magistretti P. J. (2011). Astrocyte-neuron metabolic relationships: for better and for worse. Trends Neurosci. 34, 76–87. 10.1016/j.tins.2010.12.001 21236501

[B4] Alvarez J. I. Katayama T. Prat A. (2013). Glial influence on the blood brain barrier. Glia 61, 1939–1958. 10.1002/glia.22575 24123158 PMC4068281

[B5] Araque A. Parpura V. Sanzgiri R. P. Haydon P. G. (1998a). Glutamate-dependent astrocyte modulation of synaptic transmission between cultured hippocampal neurons. Eur. J. Neurosci. 10, 2129–2142. 10.1046/j.1460-9568.1998.00221.x 9753099

[B7] Araque A. Sanzgiri R. P. Parpura V. Haydon P. G. (1998b). Calcium elevation in astrocytes causes an NMDA receptor-dependent increase in the frequency of miniature synaptic currents in cultured hippocampal neurons. J. Neurosci. 18, 6822–6829. 9712653 10.1523/JNEUROSCI.18-17-06822.1998 PMC6792963

[B6] Araque A. Parpura V. Sanzgiri R. P. Haydon P. G. (1999). Tripartite synapses: glia, the unacknowledged partner. Trends Neurosci. 22, 208–215. 10.1016/s0166-2236(98)01349-6 10322493

[B8] Ardiles A. O. Flores-Muñoz C. Toro-Ayala G. Cárdenas A. M. Palacios A. G. Muñoz P.. (2014). Pannexin 1 regulates bidirectional hippocampal synaptic plasticity in adult mice. Front. Cell. Neurosci. 8:326. 10.3389/fncel.2014.00326 25360084 PMC4197765

[B9] Bao L. Locovei S. Dahl G. (2004). Pannexin membrane channels are mechanosensitive conduits for ATP. FEBS Lett. 572, 65–68. 10.1016/j.febslet.2004.07.009 15304325

[B10] Barres B. A. (1989). Neuronal-glial interactions. A new form of transmission? Nature 339, 343–344. 10.1038/339343a0 2471078

[B11] Boucher S. Bennett S. A. (2003). Differential connexin expression, gap junction intercellular coupling and hemichannel formation in NT2/D1 human neural progenitors and terminally differentiated hNT neurons. J. Neurosci. Res. 72, 393–404. 10.1002/jnr.10575 12692906

[B13] Bruzzone S. Guida L. Zocchi E. Franco L. De Flora A. (2001). Connexin 43 hemi channels mediate Ca^2+^-regulated transmembrane NAD^+^ fluxes in intact cells. FASEB J. 15, 10–12. 10.1096/fj.00-0566fje 11099492

[B12] Bruzzone R. Hormuzdi S. G. Barbe M. T. Herb A. Monyer H. (2003). Pannexins, a family of gap junction proteins expressed in brain. Proc. Natl. Acad. Sci. U S A 100, 13644–13649. 10.1073/pnas.2233464100 14597722 PMC263867

[B14] Cherian P. P. Siller-Jackson A. J. Gu S. Wang X. Bonewald L. F. Sprague E.. (2005). Mechanical strain opens connexin 43 hemichannels in osteocytes: a novel mechanism for the release of prostaglandin. Mol. Biol. Cell 16, 3100–3106. 10.1091/mbc.e04-10-0912 15843434 PMC1165395

[B15] Chever O. Lee C. Y. Rouach N. (2014). Astroglial connexin43 hemichannels tune basal excitatory synaptic transmission. J. Neurosci. 34, 11228–11232. 10.1523/JNEUROSCI.0015-14.2014 25143604 PMC6615508

[B16] Contreras J. E. Sáez J. C. Bukauskas F. F. Bennett M. V. (2003). Gating and regulation of connexin 43 (Cx43) hemichannels. Proc. Natl. Acad. Sci. U S A 100, 11388–11393. 10.1073/pnas.1434298100 13130072 PMC208767

[B17] Contreras J. E. Sánchez H. A. Eugenin E. A. Speidel D. Theis M. Willecke K.. (2002). Metabolic inhibition induces opening of unapposed connexin 43 gap junction hemichannels and reduces gap junctional communication in cortical astrocytes in culture. Proc. Natl. Acad. Sci. U S A 99, 495–500. 10.1073/pnas.012589799 11756680 PMC117588

[B18] Contreras J. E. Sánchez H. A. Véliz L. P. Bukauskas F. F. Bennett M. V. Sáez J. C. (2004). Role of connexin-based gap junction channels and hemichannels in ischemia-induced cell death in nervous tissue. Brain Res. Brain Res. Rev. 47, 290–303. 10.1016/j.brainresrev.2004.08.002 15572178 PMC3651737

[B19] Cotrina M. L. Lin J. H. Alves-Rodrigues A. Liu S. Li J. Azmi-Ghadimi H.. (1998). Connexins regulate calcium signaling by controlling ATP release. Proc. Natl. Acad. Sci. U S A 95, 15735–15740. 10.1073/pnas.95.26.15735 9861039 PMC28113

[B20] Cotrina M. L. Lin J. H. Nedergaard M. (2008). Adhesive properties of connexin hemichannels. Glia 56, 1791–1798. 10.1002/glia.20728 18649405 PMC2577568

[B21] De Bock M. Culot M. Wang N. Bol M. Decrock E. De Vuyst E.. (2011). Connexin channels provide a target to manipulate brain endothelial calcium dynamics and blood-brain barrier permeability. J. Cereb. Blood Flow Metab. 31, 1942–1957. 10.1038/jcbfm.2011.86 21654699 PMC3185887

[B22] De Bock M. Wang N. Bol M. Decrock E. Ponsaerts R. Bultynck G.. (2012). Connexin 43 hemichannels contribute to cytoplasmic Ca^2+^ oscillations by providing a bimodal Ca^2+^-dependent Ca^2+^ entry pathway. J. Biol. Chem. 287, 12250–12266. 10.1074/jbc.M111.299610 22351781 PMC3320976

[B23] Dermietzel R. Hertberg E. L. Kessler J. A. Spray D. C. (1991). Gap junctions between cultured astrocytes: immunocytochemical, molecular and electrophysiological analysis. J. Neurosci. 11, 1421–1432. 1851221 10.1523/JNEUROSCI.11-05-01421.1991 PMC6575331

[B24] Duan S. Anderson C. M. Keung E. C. Chen Y. Chen Y. Swanson R. A. (2003). P2X7 receptor-mediated release of excitatory amino acids from astrocytes. J. Neurosci. 23, 1320–1328. 12598620 10.1523/JNEUROSCI.23-04-01320.2003 PMC6742264

[B25] Duchêne A. Perier M. Zhao Y. Liu X. Thomasson J. Chauveau F.. (2016). Impact of astroglial connexins on modafinil pharmacological properties. Sleep 39, 1283–1292. 10.5665/sleep.5854 27091533 PMC4863218

[B26] Eiberger J. Degen J. Romualdi A. Deutsch U. Willecke K. Söhl G. (2001). Connexin genes in the mouse and human genome. Cell Commun. Adhes. 8, 163–165. 10.3109/15419060109080717 12064582

[B27] Finger E. C. (2016). Frontotemporal dementias. Continuum (Minneap Minn) 22, 464–489. 10.1212/CON.0000000000000300 27042904 PMC5390934

[B28] Fiori M. C. Figueroa V. Zoghbi M. E. Saéz J. C. Reuss L. Altenberg G. A. (2012). Permeation of calcium through purified connexin 26 hemichannels. J. Biol. Chem. 287, 40826–40834. 10.1074/jbc.M112.383281 23048025 PMC3504794

[B29] Garré J. M. Yang G. Bukauskas F. F. Bennett M. V. (2016). FGF-1 triggers pannexin-1 hemichannel opening in spinal astrocytes of rodents and promotes inflammatory responses in acute spinal cord slices. J. Neurosci. 36, 4785–4801. 10.1523/JNEUROSCI.4195-15.2016 27122036 PMC4846674

[B30] Goodenough D. A. Paul D. L. (2003). Beyond the gap: functions of unpaired connexon channels. Nat. Rev. Mol. Cell Biol. 4, 285–294. 10.1038/nrm1072 12671651

[B31] Henneberger C. Papouin T. Oliet S. H. Rusakov D. A. (2010). Long-term potentiation depends on release of D-serine from astrocytes. Nature 463, 232–236. 10.1038/nature08673 20075918 PMC2807667

[B32] Huckstepp R. T. Id Bihi R. Eason R. Spyer K. M. Dicke N. Willecke K.. (2010). Connexin hemichannel-mediated CO2-dependent release of ATP in the medulla oblongata contributes to central respiratory chemosensitivity. J. Physiol. 588, 3901–3920. 10.1113/jphysiol.2010.192088 20736421 PMC3000581

[B33] Iglesias R. Dahl G. Qiu F. Spray D. C. Scemes E. (2009). Pannexin 1: the molecular substrate of astrocyte “hemichannels”. J. Neurosci. 29, 7092–7097. 10.1523/JNEUROSCI.6062-08.2009 19474335 PMC2733788

[B34] Iglesias R. Locovei S. Roque A. Alberto A. P. Dahl G. Spray D. C.. (2008). P2X7 receptor-Pannexin1 complex: pharmacology and signaling. Am. J. Physiol. Cell Physiol. 295, C752–C760. 10.1152/ajpcell.00228.2008 18596211 PMC2544446

[B35] Jeanson T. Pondaven A. Ezan P. Mouthon F. Charveriat M. Giaume C. (2015). Antidepressants impact connexin 43 channel functions in astrocytes. Front. Cell. Neurosci. 9:495. 10.3389/fncel.2015.00495 26778961 PMC4703821

[B36] Jeong W. Chung C. K. Kim J. S. (2015). Episodic memory in aspects of large-scale brain networks. Front. Hum. Neurosci. 9:454. 10.3389/fnhum.2015.00454 26321939 PMC4536379

[B37] Kang N. Peng H. Yu Y. Stanton P. K. Guilarte T. R. Kang J. (2013). Astrocytes release D-serine by a large vesicle. Neuroscience 240, 243–257. 10.1016/j.neuroscience.2013.02.029 23485803 PMC4335764

[B38] Karagiannis A. Sylantyev S. Hadjihambi A. Hosford P. S. Kasparov S. Gourine A. V. (2015). Hemichannel-mediated release of lactate. J. Cereb. Blood Flow Metab. 36, 1202–1211. 10.1177/0271678X15611912 26661210 PMC4900446

[B39] Kimelberg H. K. (2005). Astrocytic swelling in cerebral ischemia as a possible cause of injury and target for therapy. Glia 50, 389–397. 10.1002/glia.20174 15846797

[B40] Liu X. Gangoso E. Yi C. Jeanson T. Kandelman S. Mantz J.. (2016). General anesthetics have differential inhibitory effects on gap junction channels and hemichannels in astrocytes and neurons. Glia 64, 524–536. 10.1002/glia.22946 26666873

[B41] Liu X. Sun L. Torii M. Rakic P. (2012). Connexin 43 controls the multipolar phase of neuronal migration to the cerebral cortex. Proc. Natl. Acad. Sci. U S A 109, 8280–8285. 10.1073/pnas.1205880109 22566616 PMC3361458

[B42] Locovei S. Bao L. Dahl G. (2006a). Pannexin 1 in erythrocytes: function without a gap. Proc. Natl. Acad. Sci. U S A 103, 7655–7659. 10.1073/pnas.0601037103 16682648 PMC1472500

[B43] Locovei S. Wang J. Dahl G. (2006b). Activation of pannexin 1 channels by ATP through P2Y receptors and by cytoplasmic calcium. FEBS Lett. 580, 239–244. 10.1016/j.febslet.2005.12.004 16364313

[B44] Malarkey E. B. Parpura V. (2008). Mechanisms of glutamate release from astrocytes. Neurochem. Int. 52, 142–154. 10.1016/j.neuint.2007.06.005 17669556 PMC2267911

[B45] Montero T. D. Orellana J. A. (2015). Hemichannels: new pathways for gliotransmitter release. Neuroscience 286, 45–59. 10.1016/j.neuroscience.2014.11.048 25475761

[B46] Moore A. R. Zhou W. L. Sirois C. L. Belinsky G. S. Zecevic N. Antic S. D. (2014). Connexin hemichannels contribute to spontaneous electrical activity in the human fetal cortex. Proc. Natl. Acad. Sci. U S A 111, E3919–E3928. 10.1073/pnas.1405253111 25197082 PMC4169969

[B47] Moraga-Amaro R. Jerez-Baraona J. M. Simon F. Stehberg J. (2014). Role of astrocytes in memory and psychiatric disorders. J. Physiol. Paris 108, 240–251. 10.1016/j.jphysparis.2014.08.005 25169821

[B48] Nadarajah B. Jones A. M. Evans W. H. Parnavelas J. G. (1997). Differential expression of connexins during neocortical development and neuronal circuit formation. J. Neurosci. 17, 3096–3111. 9096144 10.1523/JNEUROSCI.17-09-03096.1997 PMC6573667

[B49] Nagy J. I. Patel D. Ochalski P. A. Stelmack G. L. (1999). Connexin30 in rodent, cat and human brain: selective expression in gray matter astrocytes, co-localization with connexin43 at gap junctions and late developmental appearance. Neuroscience 88, 447–468. 10.1016/s0306-4522(98)00191-2 10197766

[B50] Naus C. C. Bechberger J. F. Caveney S. Wilson J. X. (1991). Expression of gap junction genes in astrocytes and C6 glioma cells. Neurosci. Lett. 126, 33–36. 10.1016/0304-3940(91)90364-y 1650934

[B51] Nedergaard M. (1994). Direct signaling from astrocytes to neurons in cultures of mammalian brain cells. Science 263, 1768–1771. 10.1126/science.8134839 8134839

[B52] Neijssen J. Herberts C. Drijfhout J. W. Reits E. Janssen L. Neefjes J. (2005). Cross-presentation by intercellular peptide transfer through gap junctions. Nature 434, 83–88. 10.1038/nature03290 15744304

[B54] Orellana J. A. Avendaño B. C. Montero T. D. (2014). Role of connexins and pannexins in ischemic stroke. Curr. Med. Chem. 21, 2165–2182. 10.2174/0929867321666131228191714 24372216

[B55] Orellana J. A. Froger N. Ezan P. Jiang J. X. Bennett M. V. Naus C. C.. (2011a). ATP and glutamate released via astroglial connexin 43 hemichannels mediate neuronal death through activation of pannexin 1 hemichannels. J. Neurochem. 118, 826–840. 10.1111/j.1471-4159.2011.07210.x 21294731 PMC3108012

[B60] Orellana J. A. Shoji K. F. Abudara V. Ezan P. Amigou E. Saez P. J.. (2011b). Amyloid β-induced death in neurons involves glial and neuronal hemichannels. J. Neurosci. 31, 4962–4977. 10.1523/JNEUROSCI.6417-10.2011 21451035 PMC6622997

[B56] Orellana J. A. Hernández D. E. Ezan P. Velarde V. Bennett M. V. Giaume C.. (2010). Hypoxia in high glucose followed by reoxygenation in normal glucose reduces the viability of cortical astrocytes through increased permeability of connexin 43 hemichannels. Glia 58, 329–343. 10.1002/glia.20926 19705457 PMC2794960

[B57] Orellana J. A. Moraga-Amaro R. Díaz-Galarce R. Rojas S. Maturana C. J. Stehberg J.. (2015). Restraint stress increases hemichannel activity in hippocampal glial cells and neurons. Front. Cell. Neurosci. 9:102. 10.3389/fncel.2015.00102 25883550 PMC4382970

[B58] Orellana J. A. Sáez P. J. Cortés-Campos C. Elizondo R. J. Shoji K. F. Contreras-Duarte S.. (2012). Glucose increases intracellular free Ca^2+^ in tanycytes via ATP released through connexin 43 hemichannels. Glia 60, 53–68. 10.1002/glia.21246 21987367 PMC3417330

[B59] Orellana J. A. Sáez P. J. Shoji K. F. Schalper K. A. Palacios-Prado N. Velarde V.. (2009). Modulation of brain hemichannels and gap junction channels by pro-inflammatory agents and their possible role in neurodegeneration. Antioxid. Redox Signal. 11, 369–399. 10.1089/ars.2008.2130 18816186 PMC2713807

[B53] Orellana J. A. Stehberg J. (2014). Hemichannels: new roles in astroglial function. Front. Physiol. 5:193. 10.3389/fphys.2014.00193 24987373 PMC4060415

[B61] Packard M. G. Knowlton B. J. (2002). Learning and memory functions of the basal ganglia. Annu. Rev. Neurosci. 25, 563–593. 10.1146/annurev.neuro.25.112701.142937 12052921

[B62] Pan H. C. Chou Y. C. Sun S. H. (2015). P2X7 R-mediated Ca^2+^ -independent d-serine release via pannexin-1 of the P2X7 R-pannexin-1 complex in astrocytes. Glia 63, 877–893. 10.1002/glia.22790 25630251

[B63] Panchin Y. Kelmanson I. Matz M. Lukyanov K. Usman N. Lukyanov S. (2000). A ubiquitous family of putative gap junction molecules. Curr. Biol. 10, R473–R474. 10.1016/s0960-9822(00)00576-5 10898987

[B64] Pannasch U. Vargová L. Reingruber J. Ezan P. Holcman D. Giaume C.. (2011). Astroglial networks scale synaptic activity and plasticity. Proc. Natl. Acad. Sci. U S A 108, 8467–8472. 10.1073/pnas.1016650108 21536893 PMC3100942

[B65] Parpura V. Basarsky T. A. Liu F. Jeftinija K. Jeftinija S. Haydon P. G. (1994). Glutamate-mediated astrocyte-neuron signalling. Nature 369, 744–747. 10.1038/369744a0 7911978

[B66] Pellerin L. (2008). Brain energetics (thought needs food). Curr. Opin. Clin. Nutr. Metab. Care 11, 701–705. 10.1097/MCO.0b013e328312c368 18971641

[B67] Penuela S. Harland L. Simek J. Laird D. W. (2014). Pannexin channels and their links to human disease. Biochem. J. 461, 371–381. 10.1042/BJ20140447 25008946

[B68] Pini L. Pievani M. Bocchetta M. Altomare D. Bosco P. Cavedo E.. (2016). Brain atrophy in Alzheimer’s Disease and aging. Ageing Res. Rev. 10.1016/j.arr.2016.01.002 [Epub ahead of print]. 26827786

[B69] Prochnow N. Abdulazim A. Kurtenbach S. Wildförster V. Dvoriantchikova G. Hanske J.. (2012). Pannexin1 stabilizes synaptic plasticity and is needed for learning. PLoS One 7:e51767. 10.1371/journal.pone.0051767 23284764 PMC3527502

[B70] Quesseveur G. Portal B. Basile J. A. Ezan P. Mathou A. Halley H.. (2015). Attenuated levels of hippocampal connexin 43 and its phosphorylation correlate with antidepressant- and anxiolytic-like activities in mice. Front. Cell. Neurosci. 9:490. 10.3389/fncel.2015.00490 26733815 PMC4686612

[B71] Rash J. E. Yasumura T. Davidson K. G. Furman C. S. Dudek F. E. Nagy J. I. (2001). Identification of cells expressing Cx43, Cx30, Cx26, Cx32 and Cx36 in gap junctions of rat brain and spinal cord. Cell Commun. Adhes. 8, 315–320. 10.3109/15419060109080745 12064610 PMC1805789

[B72] Retamal M. A. Alcayaga J. Verdugo C. A. Bultynck G. Leybaert L. Saez P. J.. (2014). Opening of pannexin- and connexin-based channels increases the excitability of nodose ganglion sensory neurons. Front. Cell. Neurosci. 8:158. 10.3389/fncel.2014.00158 24999316 PMC4064533

[B73] Retamal M. A. Cortés C. J. Reuss L. Bennett M. V. Sáez J. C. (2006). S-nitrosylation and permeation through connexin 43 hemichannels in astrocytes: induction by oxidant stress and reversal by reducing agents. Proc. Natl. Acad. Sci. U S A 103, 4475–4480. 10.1073/pnas.0511118103 16537412 PMC1450196

[B74] Retamal M. A. Froger N. Palacios-Prado N. Ezan P. Sáez P. J. Saez J. C.. (2007). Cx43 hemichannels and gap junction channels in astrocytes are regulated oppositely by proinflammatory cytokines released from activated microglia. J. Neurosci. 27, 13781–13792. 10.1523/jneurosci.2042-07.2007 18077690 PMC6673621

[B75] Revel J. P. Karnovsky M. J. (1967). Hexagonal array of subunits in intercellular junctions of the mouse heart and liver. J. Cell Biol. 33, C7–C12. 10.1083/jcb.33.3.c7 6036535 PMC2107199

[B76] Reyes E. P. Cerpa V. Corvalan L. Retamal M. A. (2014). Cxs and Panx- hemichannels in peripheral and central chemosensing in mammals. Front. Cell. Neurosci. 8:123. 10.3389/fncel.2014.00123 24847209 PMC4023181

[B77] Roux L. Madar A. Lacroix M. M. Yi C. Benchenane K. Giaume C. (2015). Astroglial Connexin 43 hemichannels modulate olfactory bulb slow oscillations. J. Neurosci. 35, 15339–15352. 10.1523/JNEUROSCI.0861-15.2015 26586821 PMC6605489

[B78] Rovegno M. Soto P. A. Sáez P. J. Naus C. C. Sáez J. C. von Bernhardi R. (2015). Connexin43 hemichannels mediate secondary cellular damage spread from the trauma zone to distal zones in astrocyte monolayers. Glia 63, 1185–1199. 10.1002/glia.22808 25731866

[B79] Sahu G. Sukumaran S. Bera A. K. (2014). Pannexins form gap junctions with electrophysiological and pharmacological properties distinct from connexins. Sci. Rep. 4:4955. 10.1038/srep04955 24828343 PMC4021813

[B80] Santiago M. F. Veliskova J. Patel N. K. Lutz S. E. Caille D. Charollais A.. (2011). Targeting pannexin1 improves seizure outcome. PLoS One 6:e25178. 10.1371/journal.pone.0025178 21949881 PMC3175002

[B81] Schalper K. A. Sanchez H. A. Lee S. C. Altenberg G. A. Nathanson M. H. Saez J. C. (2010). Connexin 43 hemichannels mediate the Ca^2+^ influx induced by extracellular alkalinization. Am. J. Physiol. Cell Physiol. 299, C1504–C1515. 10.1152/ajpcell.00015.2010 20881238 PMC3774097

[B82] Sibille J. Pannasch U. Rouach N. (2014). Astroglial potassium clearance contributes to short-term plasticity of synaptically evoked currents at the tripartite synapse. J. Physiol. 592, 87–102. 10.1113/jphysiol.2013.261735 24081156 PMC3903353

[B83] Simard M. Nedergaard M. (2004). The neurobiology of glia in the context of water and ion homeostasis. Neuroscience 129, 877–896. 10.1016/j.neuroscience.2004.09.053 15561405

[B84] Song E. K. Rah S. Y. Lee Y. R. Yoo C. H. Kim Y. R. Yeom J. H.. (2011). Connexin-43 hemichannels mediate cyclic ADP-ribose generation and its Ca^2+^-mobilizing activity by NAD^+^/cyclic ADP-ribose transport. J. Biol. Chem. 286, 44480–44490. 10.1074/jbc.M111.307645 22033928 PMC3247979

[B85] Sosinsky G. E. Boassa D. Dermietzel R. Duffy H. S. Laird D. W. Macvicar B.. (2011). Pannexin channels are not gap junction hemichannels. Channels (Austin) 5, 193–197. 10.4161/chan.5.3.15765 21532340 PMC3704572

[B86] Stehberg J. Moraga-Amaro R. Salazar C. Becerra A. Echeverria C. Orellana J. A.. (2012). Release of gliotransmitters through astroglial connexin 43 hemichannels is necessary for fear memory consolidation in the basolateral amygdala. FASEB J. 26, 3649–3657. 10.1096/fj.11-198416 22665389

[B87] Stout C. E. Costantin J. L. Naus C. C. Charles A. C. (2002). Intercellular calcium signaling in astrocytes via ATP release through connexin hemichannels. J. Biol. Chem. 277, 10482–10488. 10.1074/jbc.m109902200 11790776

[B88] Stridh M. H. Tranberg M. Weber S. G. Blomstrand F. Sandberg M. (2008). Stimulated efflux of amino acids and glutathione from cultured hippocampal slices by omission of extracellular calcium: likely involvement of connexin hemichannels. J. Biol. Chem. 283, 10347–10356. 10.1074/jbc.M704153200 18272524 PMC2447665

[B89] Szatkowski M. Barbour B. Attwell D. (1990). Non-vesicular release of glutamate from glial cells by reversed electrogenic glutamate uptake. Nature 348, 443–446. 10.1038/348443a0 2247147

[B90] Takano T. Tian G. F. Peng W. Lou N. Libionka W. Han X.. (2006). Astrocyte-mediated control of cerebral blood flow. Nat. Neurosci. 9, 260–267. 10.1038/nn1623 16388306

[B91] Thompson R. J. Zhou N. MacVicar B. A. (2006). Ischemia opens neuronal gap junction hemichannels. Science 312, 924–927. 10.1126/science.1126241 16690868

[B92] Valiunas V. Polosina Y. Y. Miller H. Potapova I. A. Valiuniene L. Doronin S.. (2005). Connexin-specific cell-to-cell transfer of short interfering RNA by gap junctions. J. Physiol. 568, 459–468. 10.1113/jphysiol.2005.090985 16037090 PMC1474730

[B93] Weilinger N. L. Tang P. L. Thompson R. J. (2012). Anoxia-induced NMDA receptor activation opens pannexin channels via Src family kinases. J. Neurosci. 32, 12579–12588. 10.1523/JNEUROSCI.1267-12.2012 22956847 PMC6621249

[B94] Wenker I. C. Sobrinho C. R. Takakura A. C. Moreira T. S. Mulkey D. K. (2012). Regulation of ventral surface CO_2_/H^+^-sensitive neurons by purinergic signalling. J. Physiol. 590, 2137–2150. 10.1113/jphysiol.2012.229666 22411009 PMC3447156

[B95] Wilhelm F. Hirrlinger J. (2012). Multifunctional roles of NAD^+^ and NADH in astrocytes. Neurochem. Res. 37, 2317–2325. 10.1007/s11064-012-0760-y 22476700

[B96] Winship I. R. Plaa N. Murphy T. H. (2007). Rapid astrocyte calcium signals correlate with neuronal activity and onset of the hemodynamic response *in vivo*. J. Neurosci. 27, 6268–6272. 10.1523/jneurosci.4801-06.2007 17554000 PMC6672142

[B97] Yang J. Ruchti E. Petit J. M. Jourdain P. Grenningloh G. Allaman I.. (2014). Lactate promotes plasticity gene expression by potentiating NMDA signaling in neurons. Proc. Natl. Acad. Sci. U S A 111, 12228–12233. 10.1073/pnas.1322912111 25071212 PMC4143009

[B98] Ye Z. C. Wyeth M. S. Baltan-Tekkok S. Ransom B. R. (2003). Functional hemichannels in astrocytes: a novel mechanism of glutamate release. J. Neurosci. 23, 3588–3596. 12736329 10.1523/JNEUROSCI.23-09-03588.2003 PMC6742182

[B99] Zappalà A. Cicero D. Serapide M. F. Paz C. Catania M. V. Falchi M.. (2006). Expression of pannexin1 in the CNS of adult mouse: cellular localization and effect of 4-aminopyridine-induced seizures. Neuroscience 141, 167–178. 10.1016/j.neuroscience.2006.03.053 16690210

[B100] Zgaljardic D. J. Borod J. C. Foldi N. S. Mattis P. (2003). A review of the cognitive and behavioral sequelae of Parkinson’s disease: relationship to frontostriatal circuitry. Cogn. Behav. Neurol. 16, 193–210. 10.1097/00146965-200312000-00001 14665819

[B101] Zhang J. M. Wang H. K. Ye C. Q. Ge W. Chen Y. Jiang Z. L.. (2003). ATP released by astrocytes mediates glutamatergic activity-dependent heterosynaptic suppression. Neuron 40, 971–982. 10.1016/s0896-6273(03)00717-7 14659095

[B102] Zoidl G. Petrasch-Parwez E. Ray A. Meier C. Bunse S. Habbes H. W.. (2007). Localization of the pannexin1 protein at postsynaptic sites in the cerebral cortex and hippocampus. Neuroscience 146, 9–16. 10.1016/j.neuroscience.2007.01.061 17379420

